# Supplementary material for: Chlorinated Persistent Organic Pollutants (PCDD/Fs and PCBs) in Loggerhead Sea Turtles Stranded along the Central Adriatic Coast
Source: Animals (Basel). 2022 Nov 17;12(22):3177. doi: 10.3390/ani12223177 (PMC9686616; doi:10.3390/ani12223177)
Supplement: Supplementary file 1 [file animals-12-03177-s001.zip › animals-1973940-supplementary.pdf]

Chlorinated Persistent Organic Pollutants (PCDD/Fs and PCBs) in Loggerhead Sea Turtles Stranded along the Central Adriatic Coast

Table S1: Statistical comparison (Mann Whitney test) of PCDD/Fs, DL- and NDL-PCBs in fat tissue and liver of *C. caretta* by sex

matrix	Chemical class	mean_Female	mean_Male	p.value
**Fat tissue***	Sum NDL-PCB (ng g-1)	79,48	90,16	0,49
	DL-PCB (pg WHO-TE g-1)	10,55	11,00	0,57
	PCDD/Fs (pg WHO-TE g-1)	2,84	2,94	0,66
**liver****	Sum NDL-PCB (ng g-1)	14,11	16,28	0,57
	DL-PCB (pg WHO-TE g-1)	1,62	1,91	0,66
	PCDD/Fs (pg WHO-TE g-1)	0,48	0,73	0,11

* Results are expressed on a lipid wet basis.
** Results are expressed on a whole wet basis .

TabeS2: Statistical comparison (Kruskall-Wallis test) of PCDD/Fs, DL- and NDL-PCBs in fat tissue and liver of *C. caretta* by CCL range

matrix	Chemical class	CCL (cm) 31-40	CCL (cm) 41-50	CCL (cm) 51-60	CCL (cm) 61-70	CCL (cm) 71-80	p.value
**Fat tissue***	Sum NDL-PCB (ng g-1)	91,965	71,145	92,083	80,003	83,570	0,891
	DL-PCB (pg WHO-TE g-1)	11,300	8,329	12,454	10,087	10,868	0,772
	PCDD/Fs (pg WHO-TE g-1)	3,168	2,962	3,219	2,637	4,617	0,731
**liver****	Sum NDL-PCB (ng g-1)	8,075	18,335	20,200	13,193	14,393	0,737
	DL-PCB (pg WHO-TE g-1)	1,022	1,870	1,634	1,596	2,242	0,370
	DL-PCB (pg WHO-TE g-1)	1,022	1,870	1,634	1,596	2,242	0,370

* Results are expressed on a lipid wet basis.
** Results are expressed on a whole wet basis .
